# Supplementary material for: Splice-Junction-Based Mapping of Alternative Isoforms in the Human Proteome
Source: Cell Rep. Author manuscript; Available in PMC 2020 Jan 15. (PMC6961840; doi:10.1016/j.celrep.2019.11.026)
Supplement: 3 [file NIHMS1546469-supplement-3.zip › DF2/PXD0010154/Spleen-133-P06213-KTFEDYLHNVVFVPRPS.pdf]

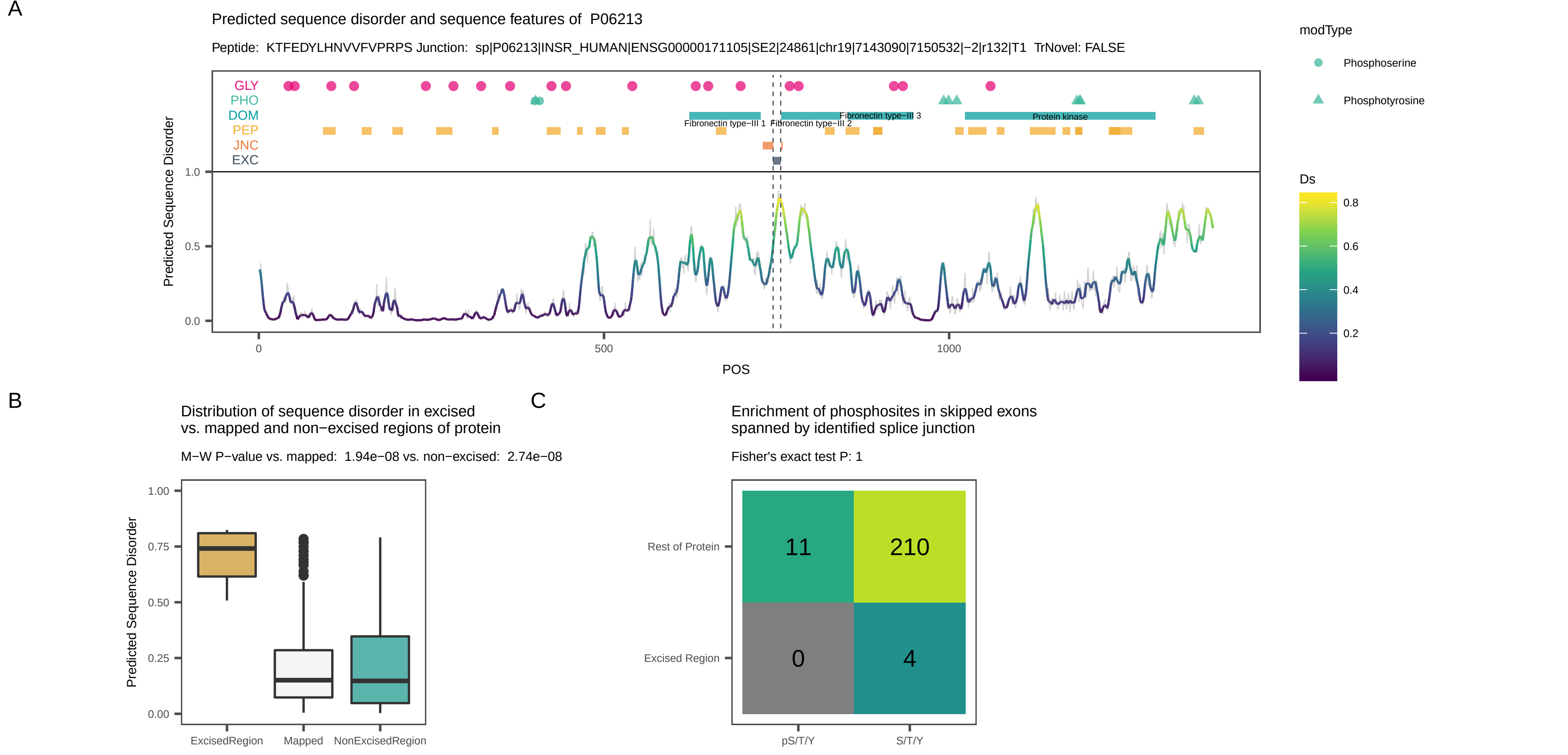

A
Predicted sequence disorder and sequence features of P06213
Peptide: KTFEDYLHNVVFVPRPS Junction: sp|P06213|INSR_HUMAN|ENSG00000171105|SE2|24861|chr19|7143090|7150532|−2|r132|T1 TrNovel: FALSE
GLY
PHO
DOM
PEP
JNC
EXC
Fibronectin type−III 1
Fibronectin type−III 2
Fibronectin type−III 3
Protein kinase
Predicted Sequence Disorder
1.0
0.5
0.0
0
500
1000
POS
modType
Phosphoserine
Phosphotyrosine
Ds
0.8
0.6
0.4
0.2
B
Distribution of sequence disorder in excised vs. mapped and non−excised regions of protein
M−W P−value vs. mapped: 1.94e−08 vs. non−excised: 2.74e−08
Predicted Sequence Disorder
1.00
0.75
0.50
0.25
0.00
ExcisedRegion
Mapped
NonExcisedRegion
C
Enrichment of phosphosites in skipped exons spanned by identified splice junction
Fisher's exact test P: 1
Rest of Protein
Excised Region
11
210
0
4
pS/T/Y
S/T/Y
